# Opioids Aware: a general practice audit of high-dose opioid prescribing NHS England Midlands and East

**DOI:** 10.3399/bjgpopen18X101642

**Published:** 2019-05-01

**Authors:** Ruth Bastable, Sarah Rann

**Affiliations:** 1 Medical Adviser, NHS England Midlands and East, Fulbourn, UK; 2 Controlled Drugs Accountable Officer and Assistant Medical Director, NHS England Midland and East, Fulbourn, UK

**Keywords:** Prescribing, analgesics, opioid, chronic pain, general practice, primary care

## Introduction

There has been a marked increase in the number of patients in the UK taking opioids from 2.5% in 2000 to 5% in 2015.^1^ More patients are taking opioids at higher doses for chronic pain,^2,3^ for which there is limited evidence of effectiveness.

A public facing^4^ audit with educational resources of high-dose opioids (≥120 mg oral morphine equivalent [OME]) prescribed for chronic pain was carried out in 74 general practices in the East of England, representing 663 418 patients.

The dose of ≥120 mg OME was chosen as doses above this are not associated with increased benefit but are associated with increased harms.^2^

The aim of this study was to quantify:

- the prevalence of high-dose (≥120 mg OME per day) opioid prescribing for chronic pain in general practices in the East of England;
- markers of prescribing quality (clear dose and frequency; clear indication; and review within the last 3 months);
- evidence of possible misuse or overuse; and
- co-prescribing of other potentially dependence-forming medications (DFMs)^1^ (such as, benzodiazepines, z drugs, gabapentin, and pregabalin).

This study also aimed to record practice reflections and plans on their results.

## Method

In total, 517 practices throughout NHS England Midland and East (East) were emailed (January 2018), inviting participation in an audit of high-dose opioids prescribed for chronic pain. Downloadable searches were provided. It was suggested that clerical staff answered the nine audit questions, and reflection should be undertaken by a GP. Participants were not offered specific advice on how the questions should be answered. No incentives were offered or directives issued.

**Figure 1.**
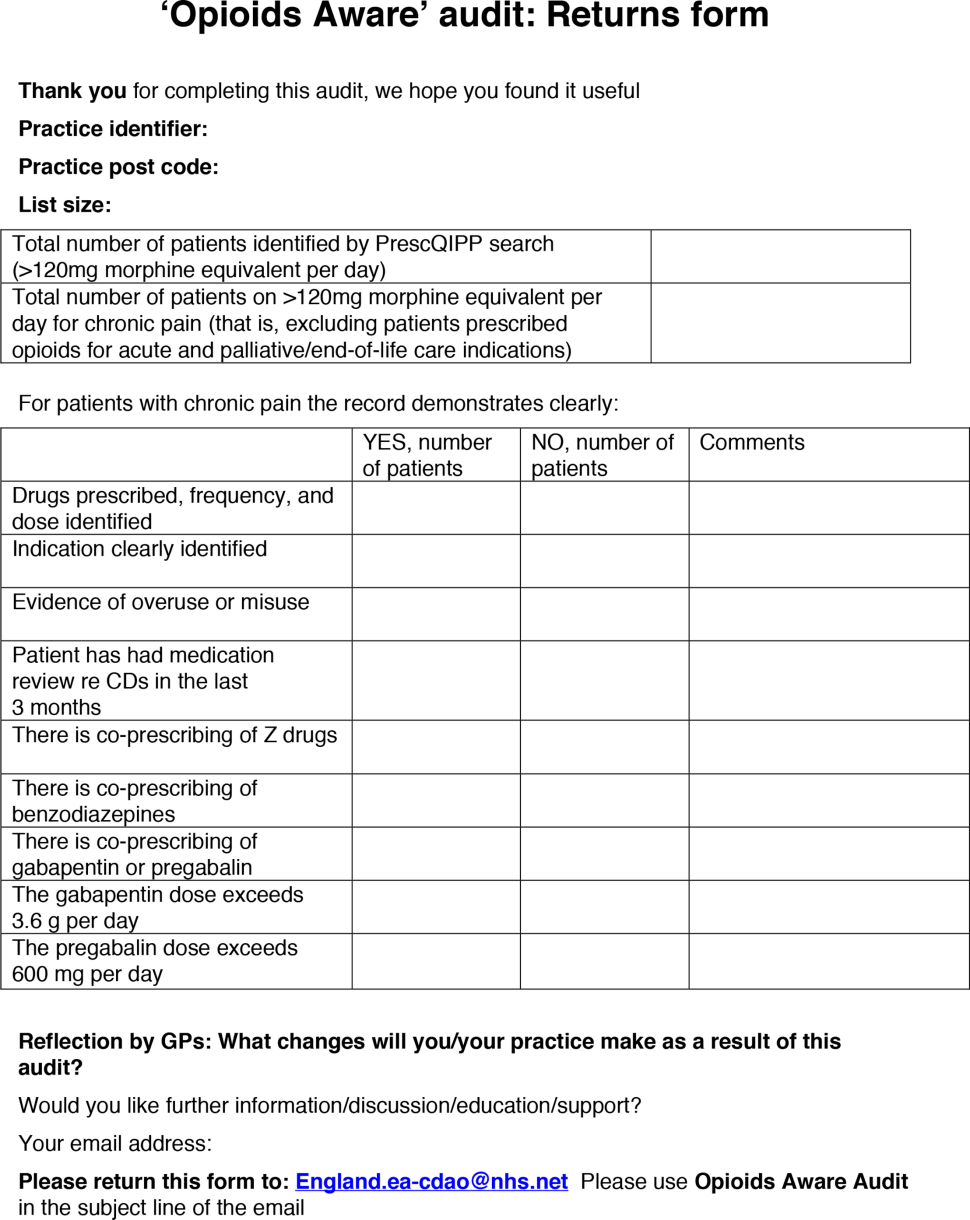
Returns form

The ‘Returns form’ (Figure 1) explained that the search identified all patients on high-dose opioids, although practices needed to manually exclude patients prescribed opioids for acute and palliative or end-of-life care indications.

The search for high-dose opioids excluded patients on buprenorphine tablets and methadone so that patients on opioids for substance misuse treatment were not included. Educational resources were also provided with web links to key references, resources, and webinars.^4^

## Results

In total, 74 practices (14.3%) responded, representing 663 418 patients (Table 1). Of these, 1022 patients were identified as being prescribed high-dose opioids (prevalence 0.15%). Almost all were for chronic pain (894/1022 = 87%, prevalence 0.13%). There was considerable variation in the number and percentage of patients prescribed high-dose opioids by practice (prevalence 0% to 0.6%) and the indication for the prescribing of opioids was unclear in 31.1%. Evidence of misuse or overuse was identified in 6.8%. Medication reviews for opioid prescribing within the previous 3 months occurred in 48.0% of patients. Co-prescribing of other DFMs was common: 13.3% for z drugs; 14.1% for benzodiazepines; and 42.4% for gabapentin or pregabalin. Prescribing outside *British National Formulary* limits for gabapentin and pregabalin for patients on high-dose opioids was uncommon (0.6% and 1.2% respectively). Eight of the 74 practices (10.8%) had no patients on high-dose opioids.

**Table 1.** Results of the audit

Patients, *n*	≥120 mg OME	≥120 mg OME for chronic pain	Drugs dose frequency	Indication	Overuse/misuse	Medication review last 3 months	Co-prescribing z drugs	Co-prescribing benzdiazepines	Co-prescribing gaba drugs	Gabapentin >3.6 g	Pregabalin >600 mg	Reflection
663 418	1022 (0.15% on high-dose opioids)	894 (0.13% on high-dose opioids for chronic pain;(894/1022 = 87.5% on high-dose for of chronic pain)	861 (96.3% clear)	593 (In 593/861 patients the indications is clear =68.9%; 31.1% not clear)	61 (61/894 = 6.8%)	429 (429/894 = 48.0%)	119 (119/894 =13.3%)	126 (126/894 = 14.1%)	379 (379/894 co-prescribed gaba drugs = 42.4%)	5 (5/894 on >3.6 g per day = 0.6%)	11 (11/894 on >600 mg pregabalin per day = 1.2%)	59 (59/74=80%)

OME = oral morphine equivalent.

In total 59 of the 74 practices provided reflections (80%).^4^ The key theme was that high-dose opioids should be treated like other high-risk drugs. Practices noted that there should be:

- clearer indication for prescribing;
- regular reviews of prescribing;
- designated GP and care plans;
- team working, up-skilling, and education of the team; and
- reduction of opioid prescribed and co-prescribing of DFMs, reflecting that this can be difficult, as can be primary–secondary care interfaces.

## Discussion

The audit has some important limitations:

1. The response rate was low, so the results cannot be generalised.
2. The number of patients on high-dose opioids may have been underestimated: the search only identified one drug at a dose of ≥120 mg OME and did not detect combinations of opioids of ≥120 mg OME.
3. Patients on buprenorphine tablets or methadone were excluded but could have been prescribed these medications for pain.
4. The percentage of patients on high-dose opioids for chronic pain is inaccurate as the indication for prescribing was unclear in many patients.
5. Participants were not asked for duration of prescribing, which would have provided additional perspective.
6. The audit questions, though not the reflection, were completed by a non-clinical member of staff and no instruction was offered regarding how misuse and overuse should be identified.

While acknowledging the limitations, it was found that the prevalence of high-dose opioids for chronic pain was low (0.13%) but variable by practice. This is in keeping with national data which shows variation by clinical commissioning group (CCG),^5^ between practices, by geography, by deprivation, rurality, and other multiple factors.^3^

Prescribing quality was of concern with respect to clarity regarding indication and frequency of review. Addressing this may have a beneficial effect on patient safety.

Evidence of possible misuse or overuse was infrequent; this may be the case, or it may be that a more detailed instruction on how to detect this would have resulted in a higher figure. The findings in this respect are broadly in line with national data.^2^

Co-prescribing of other potentially DFMs^1^ was particularly common for gaba drugs, which are known to produce potentially fatal interactions.^6–8^ Again, there is potential for improving patient safety, both by avoiding combinations of DFMs and where appropriate deprescribing.

Overall, 80% of practices provided reflections.^4^ These demonstrated learning and change, with good ideas on how to tackle the issue of high-dose opioids for chronic pain. These are potentially generalisable to the wider primary care community. Further qualitative work investigating factors related to GP prescribing would help tailor interventions to address high-dose opioid prescribing for chronic pain.

Deprescribing was noted to be difficult and should be seen in the general context of emerging evidence on how to do this.^9^ There are examples of CCG work on high-dose opioid deprescribing.^10^

With respect to what practices should do when they recognise patients on high-dose opioids for chronic pain, they should, in our view, ‘treat high-dose opioids like any other high-risk drugs’. Some practices in this audit did this, placing high-dose opioids within existing systems and processes for high-risk drugs. In conclusion, it is difficult to generalise these results due to the low response rate, however, using this audit tool to identify and review patients on high-dose opioids for chronic pain could improve patient safety.
